# Analysis of a New Shape of Test Specimen for Block Shear Impact Test

**DOI:** 10.3390/ma13071693

**Published:** 2020-04-04

**Authors:** Andrzej Komorek, Jan Godzimirski, Marek Rośkowicz

**Affiliations:** 1 Department of Aviation, Polish Air Force University, 08-530 Deblin, Poland; 2 Department of Mechatronics and Aviation, Military University of Technology, 00-908 Warszawa, Poland; jan.godzimirski@wat.edu.pl (J.G.); marek.roskowicz@wat.edu.pl (M.R.)

**Keywords:** adhesive joint, impact strength, pendulum hammer, numerical analysis

## Abstract

This paper reports and discusses an experimental comparison of metal specimens for impact strength research of adhesive connections with different shapes of the upper element. The top element of the specimen of the cuboid shape was replaced with a disc-shaped element. The experimental investigations were supplemented with dynamic numerical calculations of the tested cases. The results of the experimental studies indicate that the material applied to the produce of the top element of the block specimen deformed plastically as a result of applying the load, which further hinders the interpretation of already problematic investigation results. The numerical analysis confirms exceeding the yield point, by stresses, of the material that the specimen elements were made of. Modified specimens were characterized by only little greater repeatability of test results and greater impact strength caused by plastic deformations of the cylindrical specimen element.

## 1. Introduction

In addition to static and fatigue loads, adhesive connections in currently designed and used constructions can also be impact-loaded. An example of this type of construction is that of modern cars, in which many elements, such as door panels, bonnets, and various polymer elements, are made using adhesive bonding technology [1,2,3]. In a collision or road accident, the adhesive joints used in the structure are often impact-loaded. In order to correctly design and ensure the impact resistance of glued structures, tests and assessments of the impact strength of used joints were carried out.

In numerous cases, adhesive joints replace [4,5] or complement traditional mechanical joints [6,7]. While the matter of the static strength of adhesive bonds is quite well known, the problem of the impact strength of adhesive joints has been the subject of very few pieces of experimental research or theoretical deliberations. This is the result of insufficient assessment of test results, as well as of the lack of exact mathematical relationships that make possible an analytical determination of the impact strength of adhesive connections, based on experimental investigation. An additional difficulty is the strong dependency of the mechanical properties of the adhesives on the strain rate [8,9,10,11], because adhesives such as polymers are visco-elastic materials. The results of a compressive and tensile examination of glue bulk samples prove that their yield stress increases as the strain rate increases. [12]. Furthermore, the tensile tests of adhesives presented in [13] shows that ultimate tensile stress rises logarithmically with the load rate. The results of quasi-static and impact shear tests of tubular overlap adhesive connections prove that the shear strength increases as the strain rate rises [12].

The impact strength tests of adhesive connections are mainly conducted with original methodologies developed for the needs of their inventors [14,15]. A major concern with regard to testing impact strength of adhesive connections is the inability to compare the results obtained with different research methods, which in turn results in using the findings exclusively for the particular cases they are dedicated to.

Amongst the test methods depicted in the available publications (research on low speeds), it is possible to distinguish the three most frequently used techniques:

- Block Shear Test (BST) [16];
- Impact Wedge Peel Test (IWPT) [1,17]; and
- The method of impact shear of lap joints loaded in tension [18,19,20,21].

The investigation described in this article relates to the first mentioned method (BST), dealing with one of its aspects—the problem with the BST method is the difficulty in very precisely preparing whole series of samples with an ideal shape—in some samples, there can be a small rotation of the elements relative to each other, which results in a very large scatter of results [13,17]. The authors propose to solve the considered problem by using, in experiments, a sample with a modified element. The proposed shape of the upper element of a sample eliminates test result errors related to the possibility of the rotation of rectangular elements and ambiguous contact of the impactor with the edge of such a sample. This article presents the comparative results of experimental and numerical investigations carried out for standard and modified block specimens.

## 2. Research Methodology

One of the problems when conducting an investigation with the BST method is the quality of the geometry of the prepared specimens [22]. 

**Figure 1:**
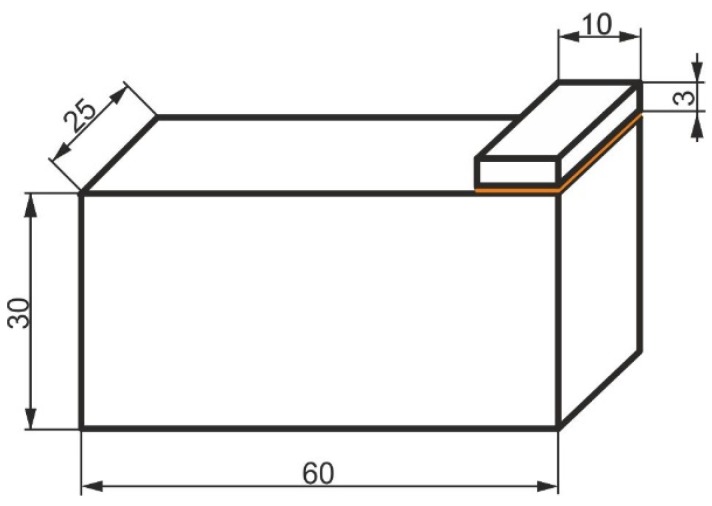
Dimensions of a block specimen (mm).

**Figure 2:**
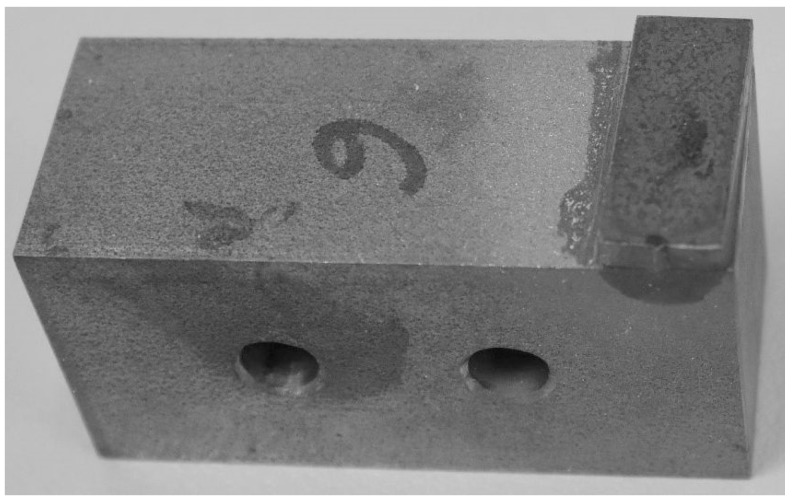
The block specimen. The top element is turned at an angle of about 1.5°.

When bonding the block specimens (Figure 1) in accordance with ISO 9653, often unintentionally, the specimen elements are slightly, almost unnoticeably rotated against each other (Figure 2). 

**Figure 3:**
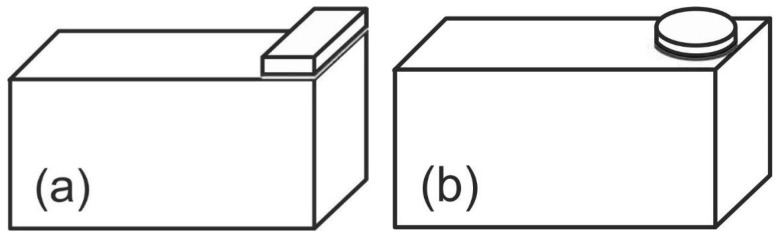
Block specimen with upper element: (**a**) according to ISO 9653; (**b**) disc-shaped.

The authors made a quantitative analysis of the impact of such distorted specimen geometry upon the registered impact strength of the examined connections [23]. The results of the numerical analyses and the experimental investigations visibly indicate major consequences of even a slight turn (even of an angle of 0.5–1°) of the specimen elements in relation to each other upon the impact strength recorded in the experiment. It should be noted that the scatter obtained in impact strength studies is large, and the above-mentioned imperfections of specimen geometry additionally increase its value, hampering or even precluding the use of research findings. The authors propose to solve the considered problem by using, in experiments, a sample whose top part, instead of being rectangular-shaped (Figure 3a), is made as a disc (Figure 3b) that can eliminate a possibility of the elements’ angular misalignment.

The specimens for performing the block shear test consisted of two metal elements: the lower one was a cuboid, whose dimensions were matched to the testing machine clamp, so that it could not move during impact loading. The other element in the first variant (Figure 3a) (in compliance with ISO 9653) was a metal cuboid, whose width equaled the width of the lower element, with the height equal to 3 mm and the width equal to 10 mm (adjusted to the maximum energy of the hammer used in the experiment). In the second variant, the top element of the specimen was a cylinder with a diameter of 17.85 mm and a height of 3 mm (Figure 3b). The surface areas of the adhesive joints in both variants were the same and equal to 250 mm^2^. 

The specimen elements were made with steel S235, which is a steel for structural purposes and is often used in different constructions. The minimum yield point of the steel was Re = 235 MPa. The surfaces of the joining elements were prepared with the method of abrasive blasting, using copper slag as the abrasive agent. The size of the copper slag particles was from 0.4 to 1.4 mm. As a result of the abrasive blasting, surface roughness with a mean arithmetic deviation of the profile from the mean line of Ra = 4.28 µm was obtained.

To connect the specimens, epoxide resin Epidian 57 with Z1 hardener was used, mixed with a 10:1 ratio (epoxy adhesive produced by chemical company “Organika Sarzyna” (Nowa Sarzyna, Poland); shear strength of not less than 15.7 MPa). The samples were glued at the same time in identical conditions, and the number of samples in each series was 10. During the putting together of the adherends for joining, special care was paid to the right setting of both elements of the specimen to each other. After putting on the adhesive, the samples were loaded with a pressure of 40 kPa. The specimens were cured for 7 days at ambient temperature (21 °C) and under steady pressure. 

After curing the adhesive in the prepared joints, a finishing treatment of specimens was performed by removing any excess of the adhesive that flew out of the adhesive layer. The thickness of the joints in all specimens was identical and amounted to about 0.1 mm. (measured with a micrometer). To ensure uniform thickness of the adhesive layer, spacer threads of a certain thickness were placed in the uncured joint. 

## 3. Findings and Discussion of the Experimental Research

**Figure 4:**
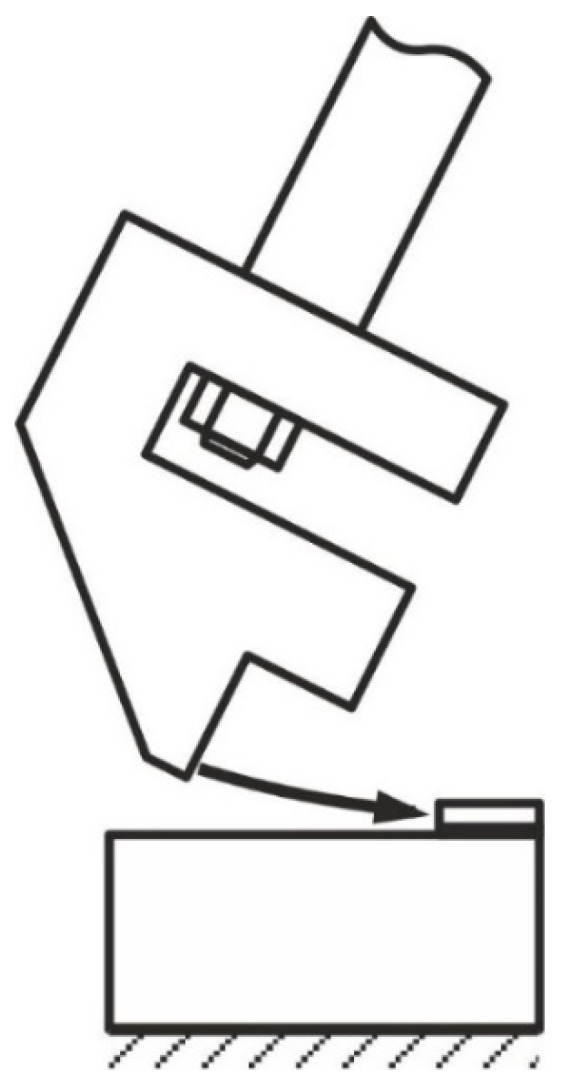
Diagram of impact strength testing in block specimens.

In this research, the load was applied to the upper part of the sample according to ISO 9653 (Figure 4) For the test results to be reliable, it was very important to keep to the conditions set out in the standard: the part of the hammer that hits the sample should be flat, wider than the element it hits, and perpendicular to it, and the bottom edge of the hammer should strike the top of the sample at a height of 0.80 mm (1/32 in) above the joint.

**Figure 5:**
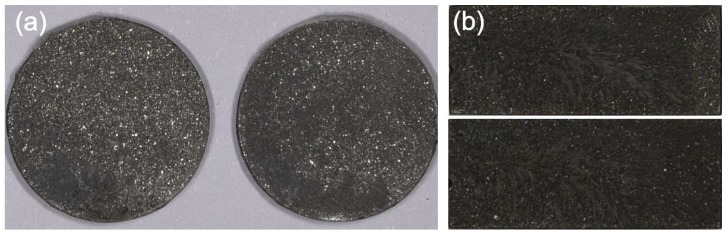
Failure of the tested joints: (**a**) upper element disc-shaped; (**b**) upper element cuboid.

The maximum impact energy used in the experiments was equal to 15 J [24] and the pendulum speed was equal to 2960 mm/s. The damage to the adhesive layers was adhesive–cohesive for both types of upper elements (Figure 5). In the specimens with a cuboid element, the percentage of the area of adhesive failures was about 40%, and in the disc-shaped elements, about 25%. In the impact tests of metals at high speeds, occurrence of phase transformations in the tested materials was demonstrated [25,26]. The impact strength tests at lower speeds showed that the nature of their damage is similar to that found in static tests.

The test results are shown in Table 1. The energy used to tear off the top element of the sample, that is, the energy lost by the hammer pendulum, was used to count the impact strength of an adhesive connection [16].

**Figure 6:**
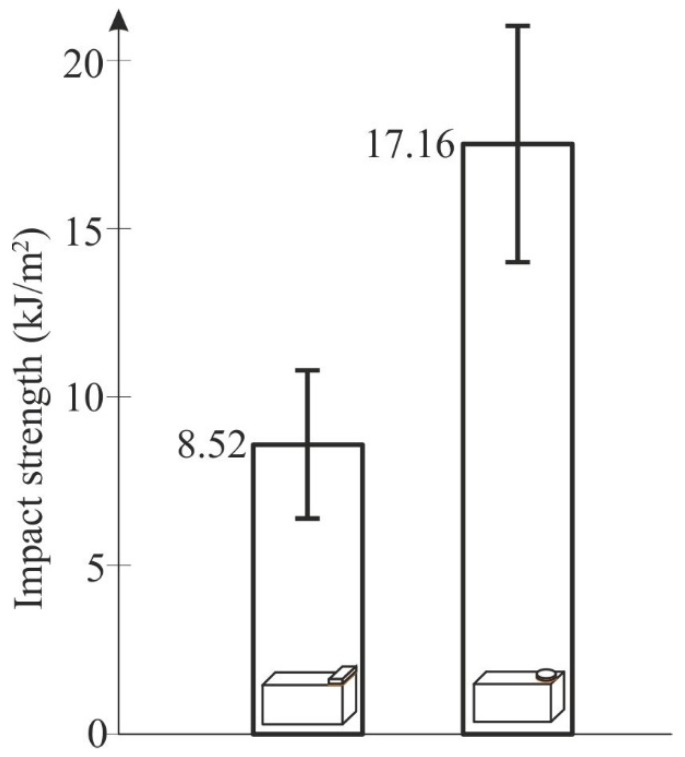
Impact strength of the specimens with top cuboid and disc-shaped elements.

**Figure 7:**
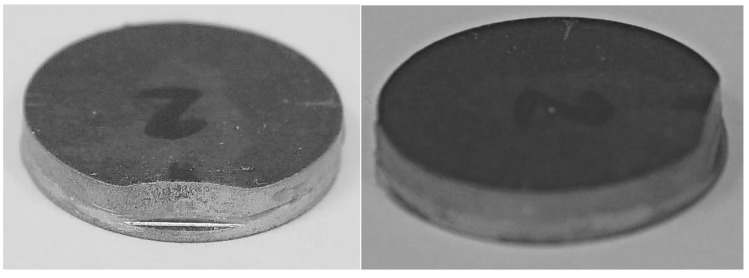
Top parts of the specimens after the impact strength tests.

The analysis of the obtained results (Figure 6) confirms the information regarding the large scatter of findings, irrespective of the top element shape, which is typical for impact tests, especially adhesive joints [16,27]. The calculated confidence interval for specimens with an upper cylindrical element was admittedly greater than that for the specimens with an upper cuboid element, but with respect to the average impact strength of both specimens, the relative value of the confidence interval for the specimens with an upper cylindrical element was 22%, and was lower than that for the specimens with an upper rectangular element (27%). With the same surface of the joints, the mean value of impact strength of specimens with cylindrical elements was twice as much as was the case in specimens where the upper element was a cuboid, which is not related to the geometry errors of the standard specimens, since prior to commencing the investigation, all of the specimens were thoroughly verified in order to eliminate the specimens with distorted geometry. The authors did not notice any plastic deformation in the specimens with cuboid top elements that were impact-loaded. However, in all of the tested specimens with a disc-shaped upper part, the impact-loaded elements underwent plastic deformation (Figure 7). The difference in the impact strength between the cylindrical and the rectangular upper elements resulted from the fact that part of the recorded energy was absorbed into the plastic deformation of the cylindrical element.

Plastic deformation of the specimens’ upper elements causes additional stress in the tested joints and deforms the research results. However, if these are comparative research (and this is the nature of block joint studies), and the elements of all of the tested specimens are distorted in the same way, then using specimens of such a shape seems to be acceptable. This statement, however, requires confirmation in additional studies. Furthermore, it is also important to perform tests in which elements of the specimens are made of a higher yield point material, as well as in which other maximum energies of the pendulum are studied, with a decreased area of the adhesive connection. Another way to eliminate plastic deformation of the top part of the sample is to use an impactor with a shape that is able to present a larger surface area to the disc.

## 4. Assumptions for Numerical Calculations

**Figure 8:**
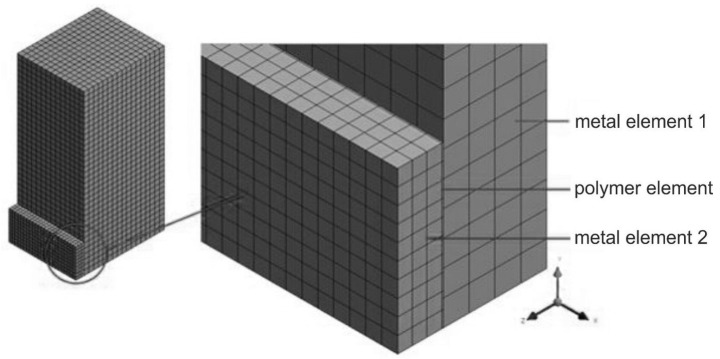
Three-dimensional (3D) model no. 1: specimen with an adhesively bonded cuboid element.

**Figure 9:**
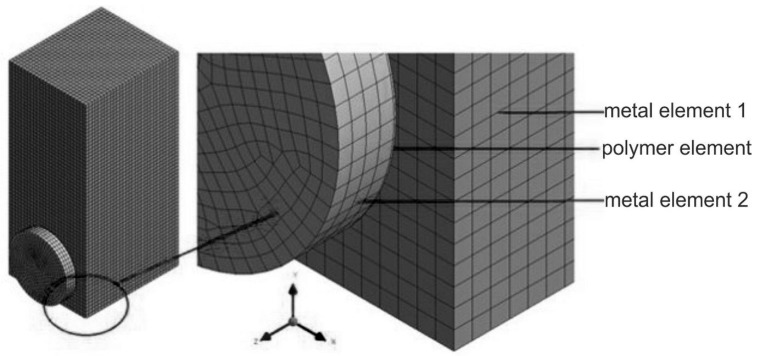
3D model no. 2: specimen with an adhesively bonded cylindrical element.

The objects of this research were two models of metal specimens, connected by adhesive joining, intended for experimental research regarding the impact strength of adhesive connections. In the numerical analysis, the properties of the standard specimens were compared, as well as the specimens where a cuboid element, which had been torn off through impact loading, was replaced with a cylindrical one of the same height and joining surface area. The models were created on the basis of the specimens used in the experimental investigations and divided into finite elements. The geometries of the specimen models exploited in numerical computations are presented in Figure 8 and Figure 9.

The dimensions adopted for the numerical computations of the three-dimensional (3D) models are presented in Table 2. During the modeling, metal elements of smaller geometric dimensions had the same volume, regardless of their shape.

In the calculations, taken into consideration were the linear features of an isotropic polymer material and the linear or nonlinear properties of the metal elements taken from the software ANSYS material database, corresponding to construction steel. The material constants for a linear range of metal elements and the polymer one, adopted for the computations, are presented in Table 3.

The numerical models were divided into finite elements, namely, 8-node hexahedral ones, to use in the dynamic calculations (impact loading). In the modeling, the bonded-type contact connection was utilized, i.e., Solid to Solid, which is usually used when glue connections are modeled.

## 5. Analysis of the Stress State in the Dynamically Loaded Glue Connections 

**Figure 10:**
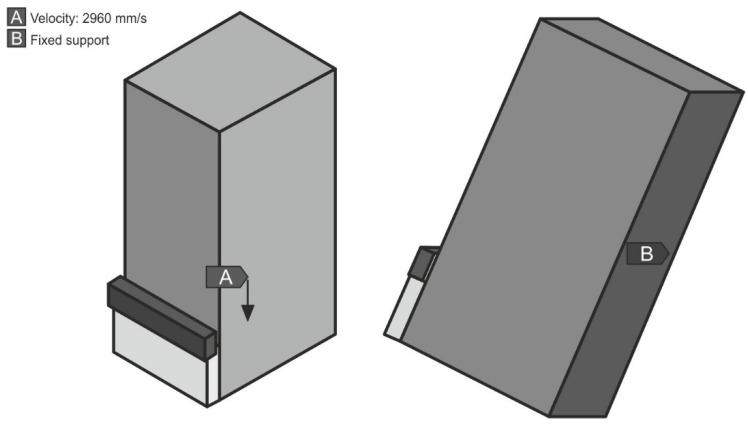
Boundary conditions: (A) speed 2960 mm/s (given to the element that simulates the hammer’s impactor); (B) surface fixing through taking away six degrees of freedom.

The models of the examined specimens were dynamically loaded with a rigid cuboid model (the authors used the rigid element) of the impactor, sized 5 × 25 × 3 mm, with a set speed of 2960 mm/s and a density of 9.2 × 10^6^ kg/m^3^, consistent with the real impact energy of the pendulum hammer, used in the tests, equal to 15 J. The loading conditions in the cases at stake were identical. The models were fixed to the surface of one of the sides of the larger metal piece through taking away six degrees of freedom. The manner of the dynamic impact loading of the specimen, as well its fixing, are presented for model 1 in Figure 10.

The initial distance of the impactor from the impacted element was 0.2 mm. The total duration of the numerical computations of the models that were loaded with the energy of the impactor equaled 10^−4^ second. The prepared models, taking into consideration the initial and boundary conditions, were subjected to dynamic numerical calculations with the use of FEM Finite element method) in the Explicit Dynamics module of the ANSYS software (v. 16.2, Canonsburg, USA). The numerical computations were done to assess the influence of the shape of the specimen on the values of the stresses occurring during the performed impact loading simulations. Based on the results of the numerical computations, the authors carried out comparative analyses of the state of the stresses in the adhesive connections with regard to parametrization of the selected boundary conditions. In the computations, it was assumed that the distance between the impactor and the adhesive layer was equal to 0.8 mm, as in the experimental tests. The maximum values of the Max Principal Stress and the von Mises Stress in the adhesive layers for the two computation times are presented in Table 4.

Figure 11, Figure 12, Figure 13, Figure 14, Figure 15, Figure 16, Figure 17, Figure 18, Figure 19, Figure 20 and Figure 21 show the distribution maps of the von Mises Stress and the Max Principal Stress values obtained in the numerical calculations. 

**Figure 11:**
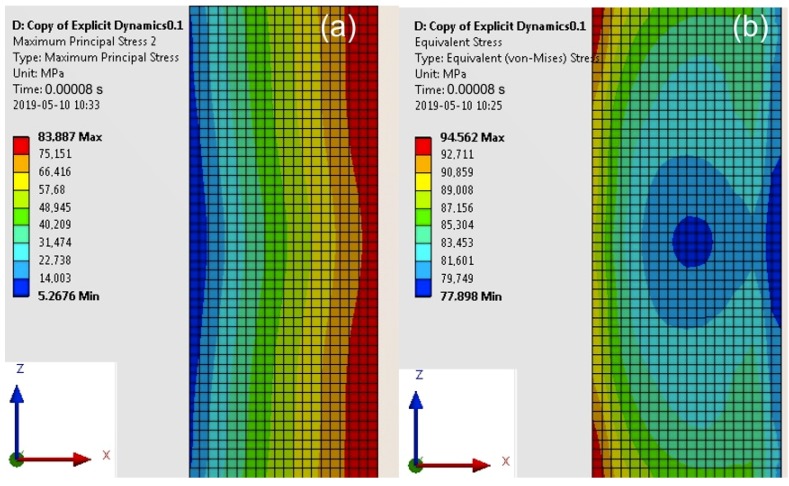
Map of the Main Principal Stress (**a**) and the von Mises Stress (**b**) in the adhesive layer for a computation time equal to 0.00008 s—Model no. 1 (linear analysis). The loads are applied in the direction of the X axis.

For specimens with a cuboid element, the maximum values of the Maximum Principal Stress occurred at the impact edge, and of the von Mises Stress at the opposite edge (similar values of the Maximum Principal Stress (83 MPa) and von Mises Stress (79 MPa) at the impact edge (Figure 11). The computation time was equal to 0.00008 s.

**Figure 12:**
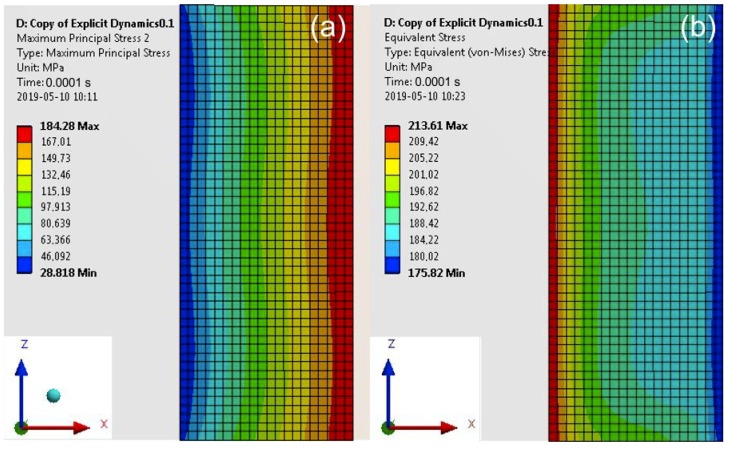
Map of the Main Principal Stress (**a**) and the von Mises Stress (**b**) in the adhesive layer for a computation time equal to 0.0001 s—Model no. 1 (linear analysis). The loads are applied in the direction of the X axis.

For a computation time equal to 0.0001 s, the maximum values of the Maximum Principal Stress occurred at the impact edge (Figure 12), and of the von Mises Stress at the opposite edge (similar values of the Maximum Principal Stress (184 MPa) and von Mises Stress (176 MPa) at the impact edge.

Taking into account the value of the breaking stress of the Epidian 57/Z1 adhesive joint, it can be assumed that the joint destruction occurred during a computational time of 0.00008 s.

**Figure 13:**
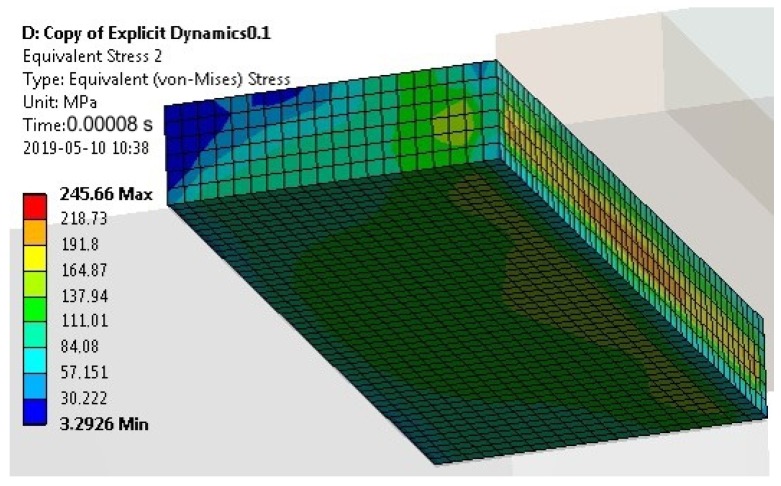
The von Mises Stress in the upper element of the specimen for a computation time equal to 0.00008 s—Model no. 1 (linear analysis).

The value of the maximum stresses in the upper element (Figure 13) slightly exceeded the yield strength of S235 steel (235 MPa).

**Figure 14:**
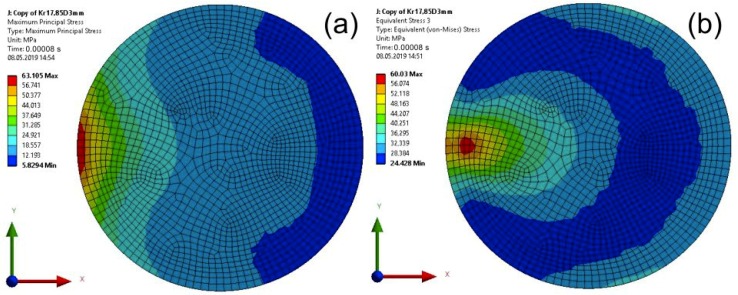
Map of the Main Principal Stress at the impact edge (**a**), and the von Mises Stress (**b**) removed from the impact edge in the joint for a computation time equal to 0.00008 s—Model no. 2 (linear analysis). The loads are applied in the direction of the X axis.

**Figure 15:**
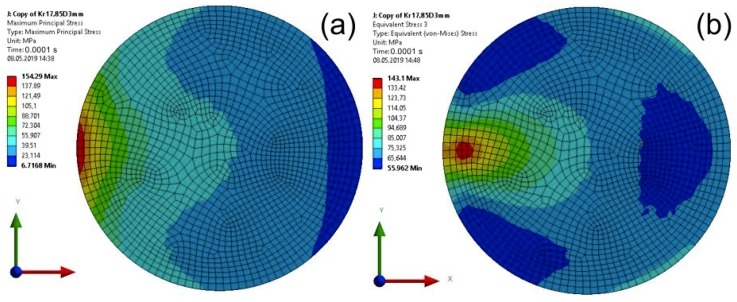
Map of the Main Principal Stress (**a**) and the von Mises Stress (**b**) in the adhesive layer for a computation time equal to 0.0001 s—Model no. 2 (linear analysis; maximal von Mises Stress is removed from the impact edge). The loads are applied in the direction of the X axis.

For specimens with a disc element, maps of the Maximum Principal Stress and the von Mises Stress are presented in Figure 14 (calculation time equal to 0.00008 s) and in Figure 15 (calculation time equal to 0.0001 s).

**Figure 16:**
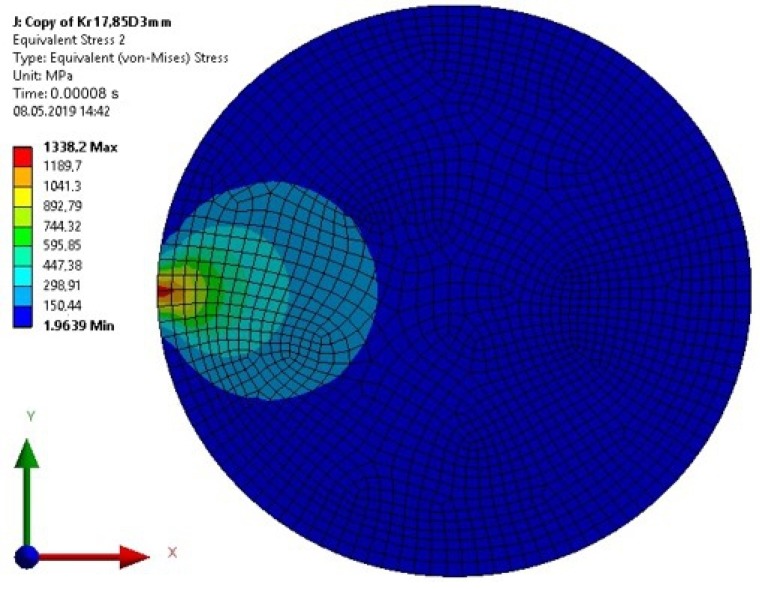
The von Mises Stress in the upper element of the specimen for a computation time equal to 0.00008 s—Model no. 2 (linear analysis). The loads are applied in the direction of the X axis.

The value of the calculated stresses in the cuboid element indicates that the yield point of the material of the top element of the specimen was exceeded for a computation time equal to 0.00008—Model 2 (Figure 16). For this calculation time, the maximum stress in the adhesive joint was less than the adhesive strength. For the calculations performed in the resilient range, the stresses in the upper element of the specimen reached unreal values.

In the following calculations, the upper element of the specimen was modeled as a plastic–elastic material with reinforcement (the plastic properties of S235 steel were taken into account). A Young’s modulus of 200 GPa, a yield point of 235 MPa, and a modulus of strain hardening of 420 MPa was declared.

**Figure 17:**
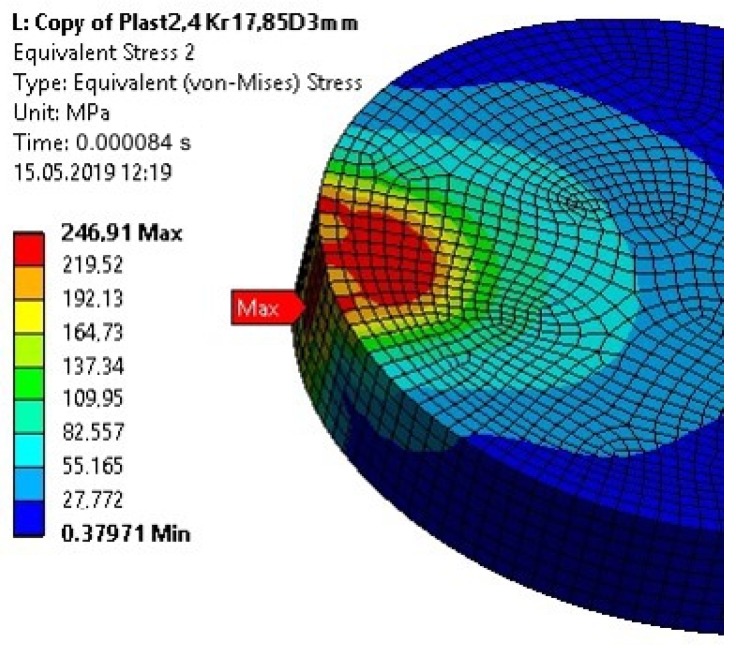
The von Mises Stress in the upper element of the specimen for the computation time equal to 0.000084 s—Model no. 2 (nonlinear analysis).

The yield point of St3 steel (S235JR) was already exceeded for a computational time of 0.000084 s (Figure 17).

**Figure 19:**
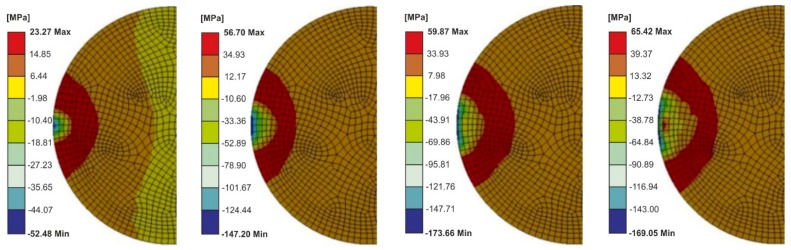

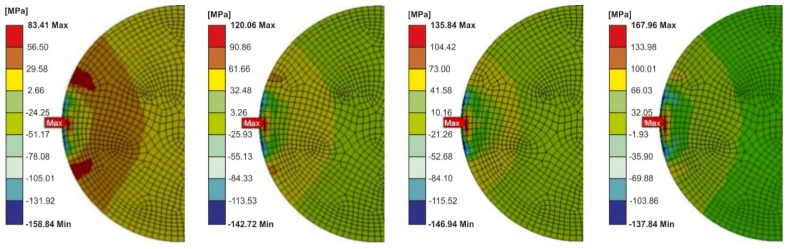
The Maximum Principal Stress in the adhesive joint for different calculations time (0.000084–0.00024 s) after taking into account the plastic properties of the upper element—Model 2 (nonlinear analysis).

The distributions of the Maximum Principal Stresses in the adhesive layer for different calculation times are shown in Figure 19.

For a time of 0.00024 (Figure 19), the maximum value of the substitute (main) stresses moved away from the adhesive layer edge, and their value exceeded the adhesive’s tensile strength (about 84 MPa).

**Figure 20:**
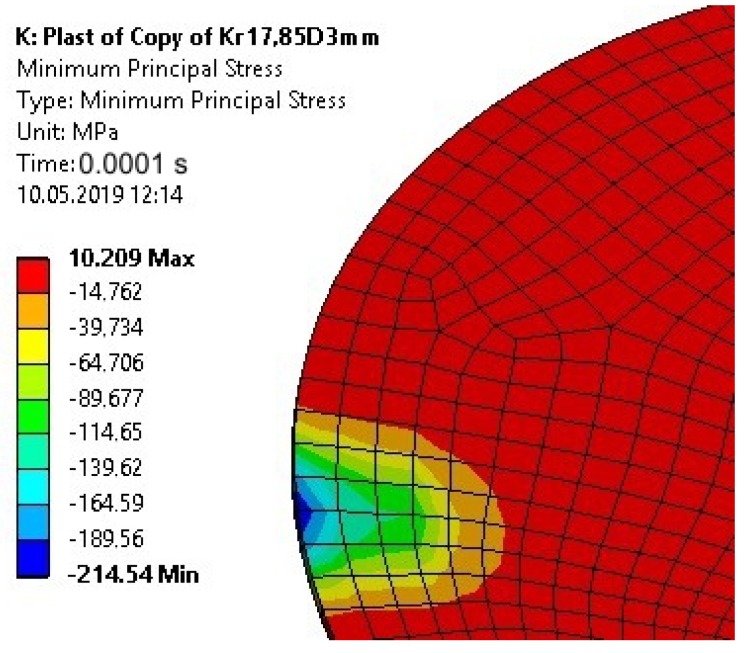
The Minimum Principal Stress in the adhesive joint for a 0.0001 s calculation time after taking into account the plastic properties of the upper element—Model no. 2 (nonlinear analysis).

The plastic deformation of the 3-mm-high disc-shaped upper element caused in the adhesive a compressive stress of high values at the edge of the joint (Figure 20).

**Figure 21:**
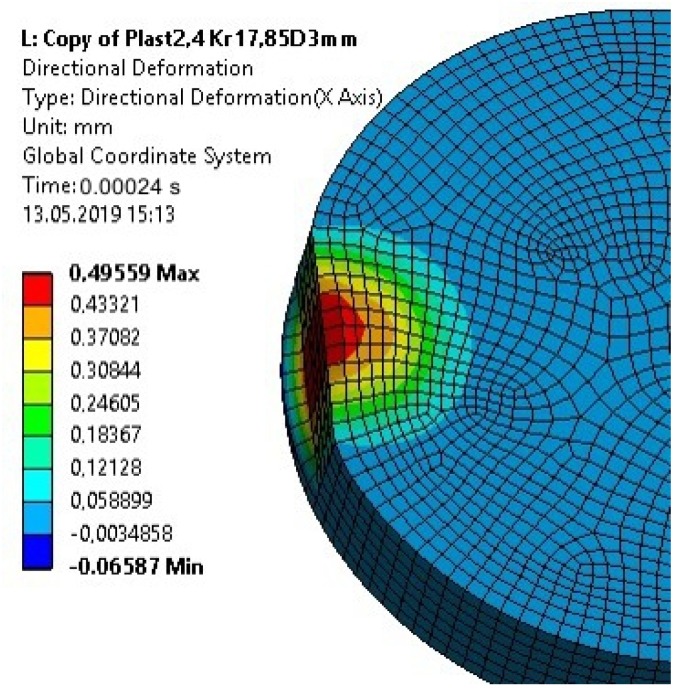
Plastic strain of the upper element of the specimen for a 0.00024 s calculation time after taking into account the plastic properties of the element—Model 2 (nonlinear analysis).

For a calculation time of 0.00024 s, the plastic deformation of the disc-shaped upper part of the specimen made of S235 steel (about 0.5 mm) was consistent with the actual deformation (from the experiment) (Figure 21).

The analysis of the Main Principal Stress values, in the joints of the investigated connections, indicates that the impact strength recorded in the investigation should be higher for specimens with an adhesively bonded cylindrical element rather than for standard geometry specimens.

**Figure 22:**
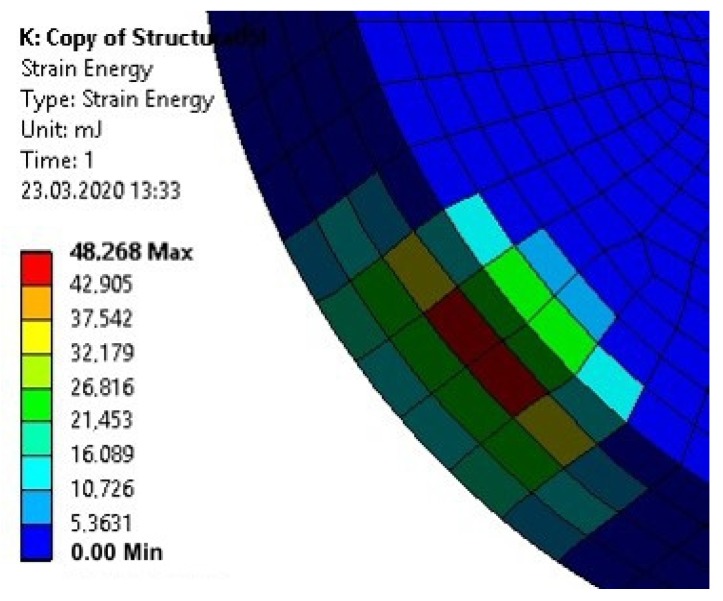
Energy of the plastic deformation of the upper element made of S235 steel loaded with 5000 N force.

**Figure 23:**
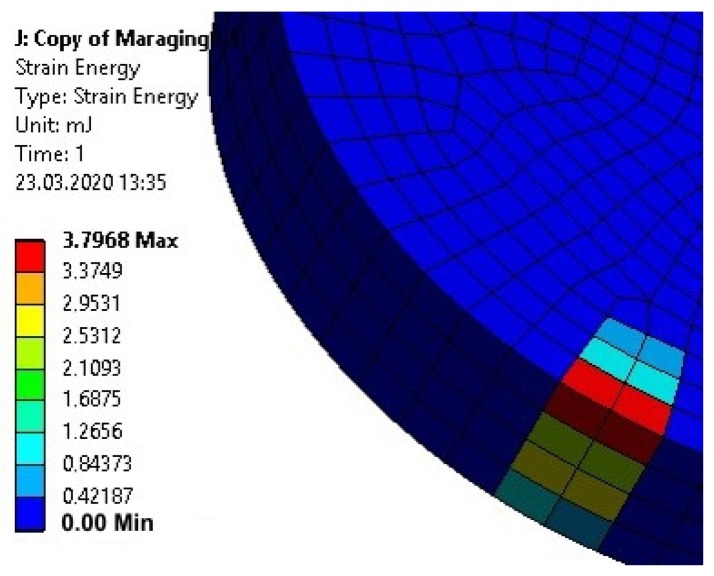
Energy of the plastic deformation of the upper element made of maraging steel loaded with 5000 N force.

The effective reduction of plastic deformation of disc-shaped upper elements would require manufacturing them from a material with a high yield point, e.g., maraging steel. Numerical calculations of the energy of deformation of disc-shaped upper elements made of S235 steel and maraging steel (with a yield point of 1500 MPa and a modulus of strain hardening of 1000 MPa) loaded with an equal force of 5000 N were conducted. The calculation results for the S235 steel element are shown in Figure 22, and for the maraging steel element in Figure 23. 

The calculation results for the maraging steel upper element showed both an order of magnitude less deformation energy in the element, as well as a smaller volume of material in which deformations occurred.

## 6. Conclusions

- The test findings obtained for specimens with a top cuboid or disc element cannot be used interchangeably, even though they have the same joining surface area.
- The modified specimens had higher impact strength due to the fact that part of the measured energy was associated with the plastic deformations of the upper specimen element.
- Because the level of stresses in the adhesively joined cylindrical metal elements exceeded the yield point of the carbon steel used in tests (235 MPa), they should be manufactured with a better quality steel, i.e., of increased yield point (for example, maraging steel where Re = 1500 MPa).
- The results of the numerical investigations are consistent with the results of the experimental research in qualitative terms; however, there is no such correlation in the quantitative aspect. The lack of correlation may be caused by disregarding the stiffness of the test machine, as well as too short a calculation time.
- The application of a disc-shaped top element in the specimen eliminates the problem of improper geometry of the specimen; however, the plastic deformation of this element during a test is undesirable, because it changes the stress state in the joint. The joint can then be damaged not only by applying impact load, but also because of the stresses resulting from the plastic deformation of the impacted element. The plastic deformation of the 3-mm-high disc-shaped upper element caused in the adhesive a compressive stress of high values at the edge of the joint.
- The decision on the usability of the specimens with a disc-shaped upper element requires further research, in which materials with significantly increased yield point should be used, as well as elements of different diameters and of higher height. The purpose of this future research will be to obtain the optimum dimensions and materials of the top element, whereby the effect of the stresses in the impacted element on the stress pattern in the adhesive joint will be minimized.

## Figures and Tables

**Figure 18:**
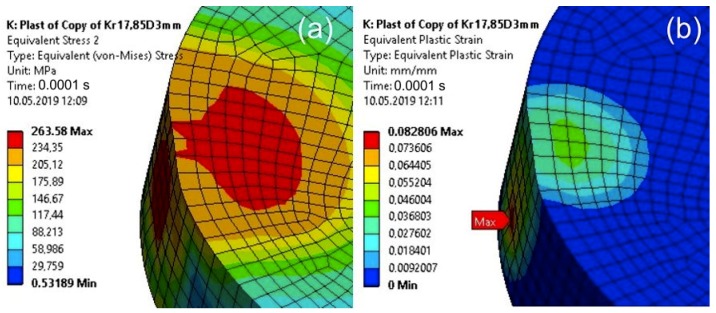
The von Mises Stress in the upper element of the specimen (**a**) and its plastic strain (**b**) for a 0.0001 s calculation time after taking into account the plastic properties of the upper element—Model 2 (nonlinear analysis).

**Table 1:** The impact test results for block specimens (the mean impact strength is given with the calculated confidence intervals for a 95% level of confidence).

Specimens According to ISO 9653 (Figure 3a)		Specimens with an Upper Disc-Shaped Element (Figure 3b)	
Impact Strength of the Joint (kJ/m^2^)	Mean Impact Strength of the Joint (kJ/m^2^)	Impact Strength of the Joint (kJ/m^2^)	Mean Impact Strength of the Joint (kJ/m^2^)
7.61	8.52 ± 2.33	12.87	17.16 ± 3.85
9.24		15.11	
13.11		15.14	
6.67		24.95	
6.03		12.11	
12.89		14.22	
4.89		12.98	
4.34		24.95	
7.89		24.94	
12.55		14.28	

**Table 2:** The geometric dimensions of the 3D models adopted for the numerical calculations.

Model No	Metal Element 1			Polymer Element (Adhesive)				Metal Element 2			
	Height	Width	Depth	Height	Width	Depth	Diameter	Height	Width	Depth	Diameter
	(mm)			(mm)				(mm)			
1	60	25	30	10	25	0.1	-	10	25	3	-
2				-	-	0.1	17.85	-	-	3	17.85

**Table 3:** Material constants of the metal elements and the polymer one (steel material data from the ANSYS library, polymer material data from own research).

Elements	Elastic Modulus	Poisson’s Ratio
	(MPa)	-
Metal 1	200 × 10^3^	0.30
Polymer	2.7 × 10^3^	0.35
Metal 2	200 × 10^3^	0.30

**Table 4:** Values of the Max Principal Stress and the von Mises Stress in the adhesive layers for different computation times (linear analysis).

Computation Time (s)	Max Stresses (MPa)	Model 1 (Specimen with a Cuboid Element)	Model 2 (Specimen with a Disc Element)
0.00008	maximum principal	83.90	63.00
	von Mises	94.60	60.03
0.0001	maximum principal	184.30	154.30
	von Mises	213.60	143.10
